# Adventures in the environment and genes

**DOI:** 10.1007/s10654-020-00604-9

**Published:** 2020-02-04

**Authors:** David J. Hunter

**Affiliations:** 1 Richard Doll Professor of Epidemiology and Medicine, Nuffield Department of Population Health, Big Data Institute, University of Oxford, Room 27, 2nd Floor, Old Rd, Oxford, OX3 7FZ USA; 2 Vincent L. Gregory Professor of Cancer Prevention, Emeritus, Harvard T.H. Chan School of Public Health, Boston, USA; 3 Professor of Medicine, Emeritus, Harvard Medical School, Boston, USA

I was greatly honored by the invitation to give the Cutter Lecture, and following the practice of some other Cutter Lecturers I would like to offer some reflections on my career in Epidemiology, and draw some lessons about best practices that I wish I had known 35 years ago when I first considered this career. I will mention along the way some mentors and colleagues, but I apologize in advance to the many I will not name who have my gratitude and respect.

Like many Australian physicians of the era, I was first properly exposed to population health during elective term experiences in Papua New Guinea, and subsequently in Dar-es-Salaam, Tanzania. After some clinical training, I enrolled in the MPH program at HSPH, vaguely expecting to wind up working for an NGO in a refugee camp. I had never really thought much about research, although an interest in trekking in the Himalayas and some minimal mountaineering experience had me reading papers on altitude sickness, and resulted in a co-authored review in the 1984 Christmas edition of the Medical Journal of Australia [1]. It was readily apparent that altitude sickness resulted from a combination of extreme environment, and inter-individual susceptibility, so I suppose the die was cast for a career investigating the inter-individual variation of environmental response.

My thesis in the Nurses’ Health Study was on risk factors for non-melanoma skin cancer, again a combination of environmental exposure and susceptibility. Using the relatively crude graphics programs of the time, the papers featured a series of three-dimensional plots showing the relation between the two (Fig. 1) [2]. The Nurses’ Health Study held (and still holds) a weekly meeting of faculty, students and staff that were a model of collaborative development of questionnaires, grants and analysis plans. I probably learned as much about Methods in those meetings as I did in the classroom.

**Fig. 1:**
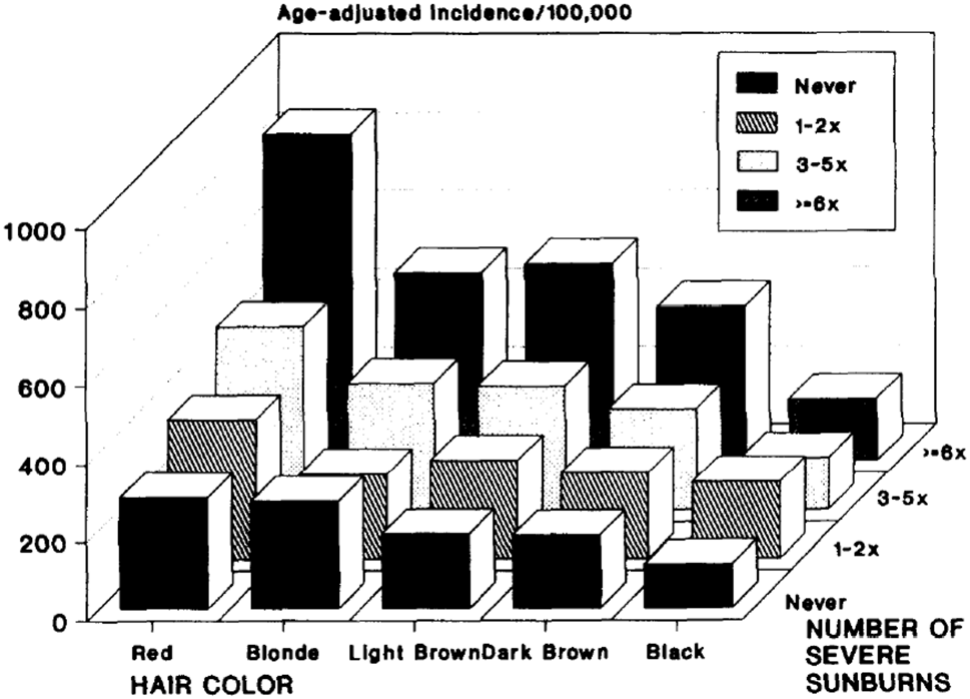
Interaction of natural hair color (inherited) and lifetime number of severe sunburns (sun exposure and susceptibility) in the Nurses Health Study using the crude graphics packages of 1988

My interest in what was then called International Health was still present, and towards the end of my doctorate I worked with Lincoln Chen as Executive Director of the AIDS and Reproductive Health Network. We developed a series of studies on HIV prevalence and incidence, and I collaborated with colleagues in Kenya and Tanzania examining HIV risk factors among women [3, 4]. We found to our horror that the prevalence was much higher than expected, meaning that studies initially designed as cross-sectional screens designed to facilitate case-control studies could be analyzed using prevalence risk ratios and then turned into prospective studies with annual incidence rates of several percent. Laptop computers had been recently introduced, and I spent many happy evenings in Nairobi and Dar-es-Salaam watching logistic models that would now converge in seconds gradually converging over hours, all the while hoping the electricity did not suddenly fail. A blackout would crash the program; a power surge might fry the laptop. I still recommend some international work to anyone early in their career who has the freedom to experience it.

Meanwhile, a project had been rather grudgingly funded by the site visitors to Walter Willett’s otherwise enthusiastically received Program Project, and we assembled an all-star cast of nutritional epidemiologists who were willing to share data post-publication, initially on diet and breast cancer. This became the Pooling Project of Prospective Studies of Diet and Cancer. In retrospect, we should have had a nifty acronym and logo, but consortium acronyms were not yet de rigueur. It was a great way to work with an international cast of epidemiologists and thus hear a diversity of opinions and approaches. We published a string of largely null papers [5] with the exception of a pooled analysis of alcohol and breast cancer [6]. For our annual meetings we would assemble a set of data tables the size of a phone book, and spend a day rather mind-numbingly working through them. Significant tests for heterogeneity were very rare, but study-specific significant relative risks not uncommon—and it was only seeing these as outliers among otherwise null findings and a null pooled relative risk that brought home to me the message of the play of chance. In essence we were learning the desirability of replication to weed out false positives, at the expense of study-specific findings that would make a good, and publishable, story.

Of course, there is still much skepticism about nutritional epidemiology. It is possibly the branch of our discipline that has the most potential for false positives because of the number of foods, nutrients, diseases, and potential interactions, combined with multiple studies often with small sample sizes. A common interpretation—that diet is too complex for people to report—does not seem justified by the development of the field and its successes in defining dietary risks for heart disease and diabetes. It is perhaps ironic that much of the work in nutritional epidemiology in the US was funded by the National Cancer Institute as the consensus finding is that if toxic agents such as aflatoxin and arsenic are excluded, there is not a strong connection between diet in middle life and the short-to-medium term risk of most cancers [7].

Reinforcement of the need for replication would come with the second grant—that funded a nested case-control study of plasma pesticide levels and breast cancer in the Nurses’ Health Study. A small, apparently exemplary, study in the New York University Women’s Health Study cohort had reported an association [8]. We failed to replicate this in the Nurses’ Health Study [9] and then in a collaboration of four other nested case-control studies [10]. Again, the lessons were that collaboration, to increase sample size and assess the reproducibility of findings was essential.

John Cairns, a Professor of Cancer Biology at HSPH, had been on my doctoral thesis committee, and was kind enough to engage in mechanistic discussions of carcinogenesis, amplifying the material in his lectures that were delivered with chalk and multiple blackboards, before the days of Powerpoint made giving a lecture rather less spontaneous. I decided to switch focus to genetics.

Starting with a small lab in the basement of the Channing Laboratory funded by Frank Speizer and collaborating with Karl Kelsey, we began to extract DNA’s from the Nurses’ Health Study buffy coats, and experimented with collecting cheek cell swabs from women who had not given blood samples. Genotyping was painfully slow using restriction fragment length polymorphisms generated by cutting with restriction enzymes and running fragments on gels. A good day’s work for a technician was a single genotype on two 12-lane gels from 24 women, but it all seemed quite high-tech. Most of my elders and betters considered this a waste of an epidemiologist’s time—lab work could be farmed out to multiple laboratories who would often do the work “for free”. Immersion in the lab world did help the studies however, notably in reducing the amount of DNA we needed, and understanding the quality control issues of genotyping. I would recommend that anyone using laboratory results at least visits the laboratory in question, but better still embeds for some time.

The real problem was that we were testing “hypotheses”, often without any idea whether the RFLP was generated by a functional variant in the gene. The genes were selected from “pathways” that were thought to be relevant to the disease under study, so-called “candidate genes”. The usual metaphor is that we were “looking for our keys under the lampposts in a darkened street”, but looking for needles in a haystack is a more apt description. Nevertheless, we acquired the ability to extract, store and reliably archive DNA samples, and a familiarity with the concepts and language of the genetic epidemiology of the time. At the time there were still many prominent geneticists who maintained that it was hopeless to study disease genetics outside family-based designs, and that conventional case–control studies would never have any place in human genetics.

By and large, we did not find many genetic main effects that were reproducible, and we were mainly involved in publishing refutations of previously published, and probably publication-biased “positive” studies. Exceptions included a small handful of functional variants for example in MTHFR and colorectal cancer [11], MC1R and skin cancer [12], and PPAR gamma and diabetes [13].

The other misapprehension under which we labored was that there would be many gene-environment interactions to discover, by which we meant situations in which the joint effect of gene and environment led to synergy and the effect of the environmental factor was much stronger in, or even restricted to, a specific genotype. To be fair, this was the experience of animal studies, and some human paradigms such as the “inborn errors of metabolism” (a phrase coined by Archibald Garrod in 1908). The search for gene-environment interactions led to a burst of funding, as well as prioritization of cohort studies in which, of course, the environmental factor could be measured more reliably than in case-control studies. Spearheaded by Bob Hoover of the NCI intramural program, cohort consortia were assembled to assess reproducibility and to attempt to have adequate power for interactions and I became co-chair with Elio Riboli of the NCI Breast and Prostate Cancer Cohort Consortium (BPC3—acronyms now being mandatory). Rather than analyzing individual level data from previously published studies, we were now pooling data prior to publication, a much more efficient and expedient way of getting to the summary results.

Meanwhile a revolution in genotyping technology was occurring. Machines had been introduced that turned the 12-lane gel into 24, then 48, then 96, then 394 sample formats, but this was scaling up one variant at a time. In about 2005 new “single-nucleotide polymorphism” (SNP) chip technologies became available that could measure first, 100,000, then 300,000 or more variants from a single DNA sample. These numbers enabled “genome-wide association studies” (GWAS), essentially lighting up the street with 300,000 lamps to increase the probability of finding our keys. SNP chips were expensive at first (over $1000 each) so studies were split into discovery (using the SNP chip) and replication studies (taking the small number of significant “hits” into larger samples. A twist of fate led to the design, along with Stephen Chanock of the NCI intramural program, of the CGEMS (Cancer-Genetic Markers of Susceptibility) program, mainly composed of studies from the BPC3. We co-discovered, along with a group of mostly case–control studies led by Doug Easton [14], the strongest common genetic variant associated with breast cancer [15] (Fig. 2), going on to other discoveries in breast and prostate cancer. Within three years we could show that the “genetic risk score” (now called polygenic risk score or PRS), derived by adding up the number of risk variants a women carried, outperformed the previous “clinical” risk scores derived from the “classical” breast cancer risk factors [16].

**Fig. 2:**
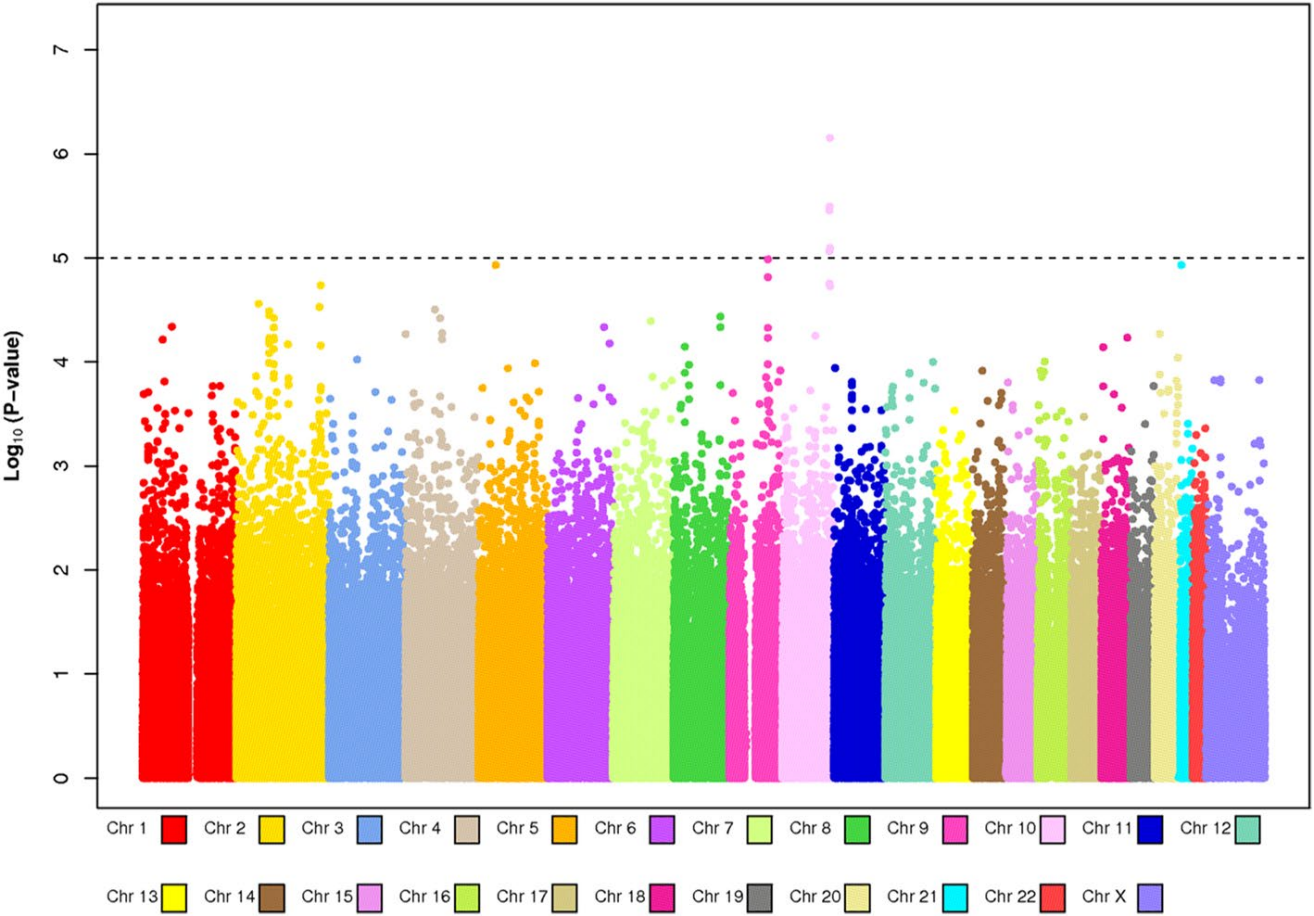
“Manhattan Plot” from the first CGEMS whole genome scan of approximately 500,000 DNA variants and breast cancer risk with smaller *P* values represented higher up on the Y axis. The smallest P values were in the FGFR2 gene on chromosome 10

What we had not appreciated was that the established pecking order of epidemiological study designs i.e. cohort studies were superior to case–control studies, did not apply to the genetic epidemiology of main effects. Robustly estimating small relative risks is a matter of power, and thus sample size, and therefore a large series of case control studies was ultimately more informative than smaller nested case-control studies. Indeed, large case series could be compared with “public” controls derived from different studies with relative safety once methods were developed to statistically control for any subtle differences in population structure. Since there were many more case series and case-control studies available, the case-control consortia soon out-performed the cohort consortia (albeit it was always reassuring when there was no heterogeneity between the case-control meta-analyses and the cohort meta-analyses). This work has proceeded in large international consortia, and there are now over 180 robust genetic variants of weak effect associated with breast cancer. Collectively, when combined in a PRS, these lead to relative risks of approximately three-fold for women in the top five percent of the risk distribution compared with the middle quintile, and six-fold for the top versus the bottom five percent [17]. Along the way, Epidemiology provided some of the key evidence that overturned the dogma that inherited variation was principally due to variants in DNA that altered the structure of a protein. Many of the GWAS hits were in intergenic regions or “gene deserts”, previously called “junk DNA”. The biologists thought we were doing something wrong, but the discovery of “pervasive transcription” i.e. that much of the DNA outside protein coding regions was transcribed into RNA suggested the functional importance of these intergenic regions, now assumed to be controlling the developmental coordination and expression of genes.

But what of the interaction paradigm that average genetic risks varied substantially according to exposures (and vice versa, that environmental effects concealed a spectrum of highly susceptible to non-susceptible people)? In brief, these have been vanishingly rare for most chronic diseases. In the BPC3 none of the tests for multiplicative interaction between classical breast cancer risk factors and the increasing number of GWAS hits survived correction for multiple comparisons [18]. This is the story for most other cancers and for heart disease and diabetes. By and large, the common genetic factors and environmental relative risks simply multiply together, without cancelling each other out, or giving evidence of supra-multiplicative synergy. This has been disappointing to those of us trained to hunt for statistical interactions on the multiplicative scale, however, interactions on the additive scale are many, thus, there is still clinical and public health relevance to the joint consideration of genes and environment. The absence of synergy actually simplifies life with respect to risk prediction as we do not often have to always account for unusually susceptible or resistant individuals.

Some of this story matured while I was taking a time-out from full-time research as Dean for Academic Affairs at the Harvard School of Public Health (now the Chan School), focused on faculty development, our strategic research plan, and how to take advantage of the new opportunities offered by Massive Online Open Courses. I was fortunate that Peter Kraft took over the work and pushed it forward. It also left me somewhat freer from the SNP by SNP discovery effort to consider the wider implications, and the state of epidemiology in general. Interestingly, despite the focus in modern genetic epidemiology on gathering large numbers of cases and controls of the various diseases, very few of the most successful groups have been led by epidemiologists. The key skills are organization of consortia, managing complex data transfer agreements, keeping up with and interpreting the latest gene chip technology, development of statistical methods to handle millions of data points, and interpreting the results in the light of the rapidly evolving knowledge of genome structure and function. Thus, knowledge of bioinformatics and genomic biology became more important than classical epidemiological skills. Many epidemiologists did acquire this cross-training of course, and I would like to think the Program on Genetic Epidemiology and Statistics in the HSPH Epidemiology Department contributed to this. However, this was a new world where hypotheses were a distraction, “agnostic” analyses triumphed, and sample size supplanted study design.

It is notable that, with the exception of studies based in administrative databases such as Medicare, epidemiology has not made the more than seven orders of magnitude leaps in throughput that genetics has, or that computers have. There has been no Moore’s Law for epidemiological sample sizes. Indeed, since Doll and Hill put together their study of 24,389 male doctors using index cards to sort lung cancer cases and controls by smoking status, the largest prospective studies are less than two orders of magnitude larger (Fig. 3). The question for epidemiology is whether we can construct larger, but still rigorous, studies, using modern technologies. In previous prospective cohorts we had the choice of small studies with a lot of detail, or large studies with skimpy detail. Can we construct large studies with even more detailed exposure information, potentially including ‘omic analyses at scale?

**Fig. 3:**
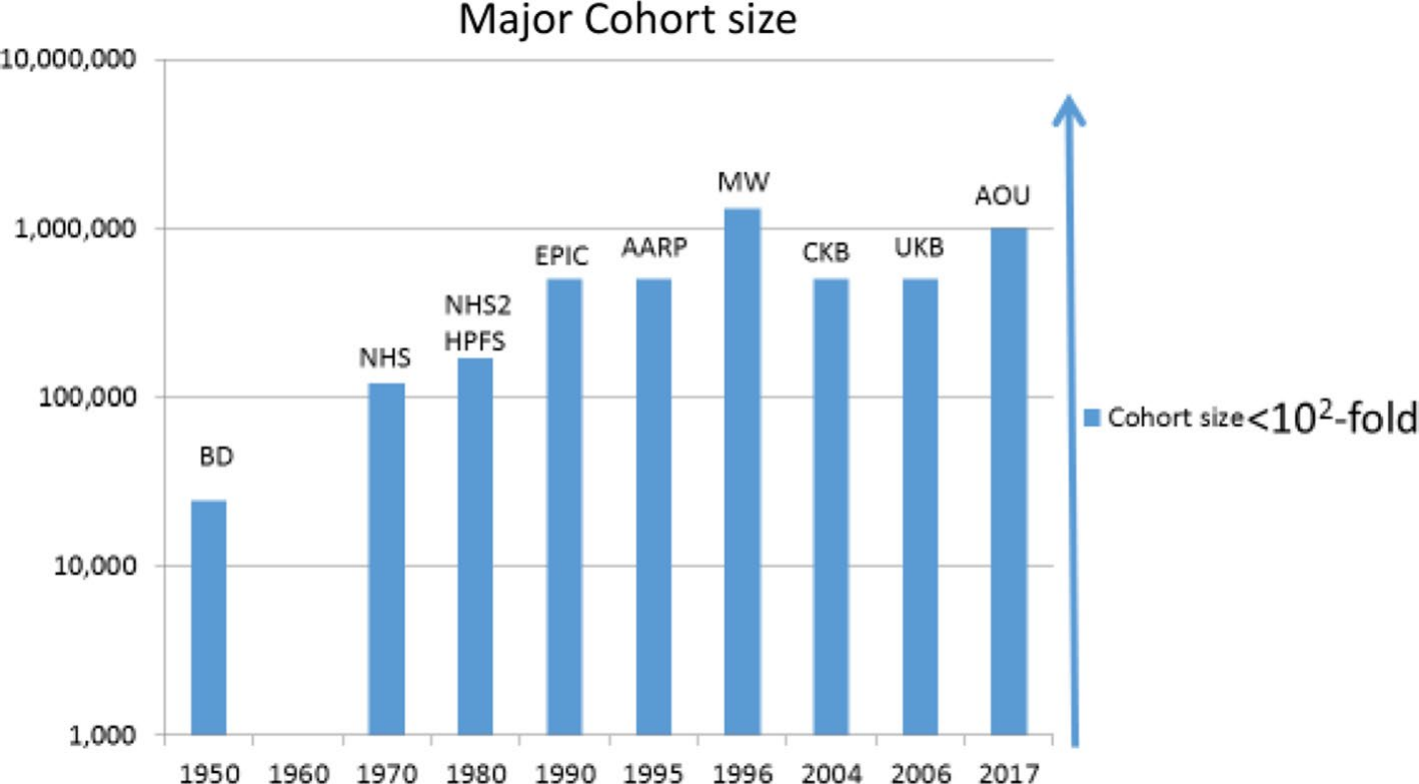
Approximate sample sizes of some of the major prospective cohort studies on a log scale. BD = British Doctors study, NHS = Nurses Health Study, NHS2 = Nurses Health Study 2, HPFS = Health Professionals Study, EPIC = European Prospective Investigation into Cancer and Nutrition, AARP = American Association of Retired Persons Diet and Health study, MW = Million Women Study, CKB = China Kadhoorie Biobank, UKB = UK Biobank, AOU = All of Us Research Program (proposed sample size)

After as a final year as Acting Dean at the Chan School I took a year’s sabbatical then moved to Oxford as the Richard Doll Professor with the intention of contributing to development of a large prospective studies in the context of the National Health System in which some of the hard work of ascertaining and validating disease diagnoses it done for us. The exemplar, of course, is the UK Biobank of 500,000 UK residents now followed for over ten years. The next step is to approximate “whole-country” prospective studies integrating self-reported exposures, primary care information, genetics, and disease outcomes supplemented by data from geographic information systems and environmental monitoring. This has been pioneered at a smaller scale in the Scandinavian countries The smartphone gives us the means to ask people about themselves remotely, and perhaps, with their consent, to monitor physical activity, geolocation, and other factors such as heart rate and cognitive function. In the 20th Century the tradeoff was almost always between having a large sample size with little individual information. In the 21st Century we should be able to combine large sample sizes with much richer exposure assessment (Fig. 4). In my Inaugural Lecture at Oxford I posed the question “Is Bigger Epidemiology Better Epidemiology?” The answer, of course, is “not always”, but it is notable that for the rare diseases we still have limited power even in consortia of cohorts, and to address risk factors for these we still need much larger studies. Finally, the PRS give us the means to identify people at high risk of many common diseases early in adult life—can we usefully intervene to lower risk in these large studies, combining observation and intervention?

**Fig. 4:**
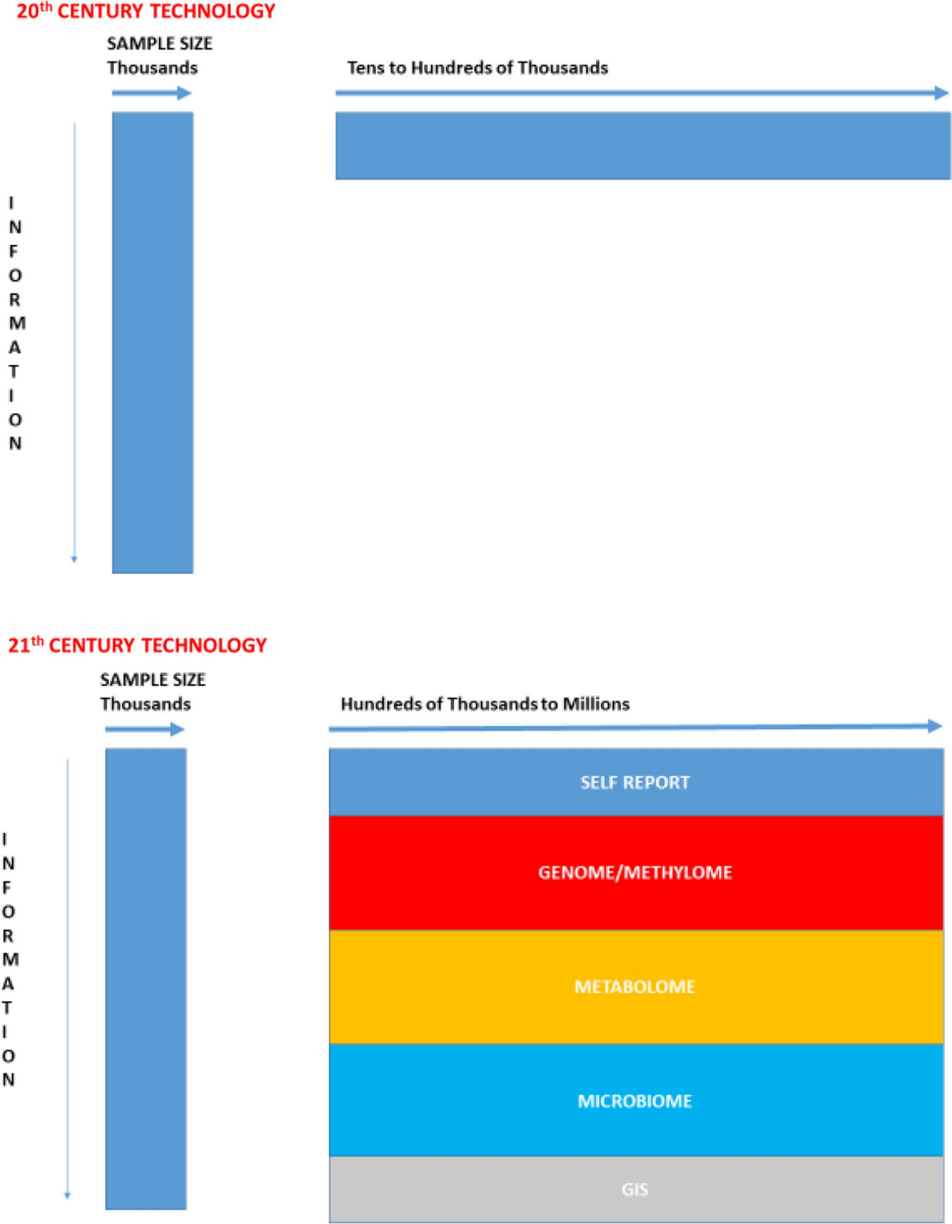
Comparison of potential prospective cohorts using 20th Century and 21st Century technologies

In a sense this is returning Epidemiology to its roots. John Snow’s study base was almost the entire population of London (or at least the fraction that drew its water from the Thames). Whether or not taking the handle off the pump made a difference, he used his observations to intervene. Since Doll, epidemiologists have discovered a wide range of environmental risk factors for many diseases. In the last decade tens of thousands of genetic variants have been associated with common diseases and other phenotypes. We have begun to understand how exposure and inherited risk intersect. New tools are becoming available that permit us to scale up our studies and follow participants at low costs. The challenge is to integrate this information into clinical and public health practice in manner that promotes health. The Adventure continues.

## References

[1] Smart J, Hunter D (1984). Alpine travel Mountain sickness, the unwelcome companion. Med J Aust..

[2] Hunter DJ, Colditz GA, Stampfer MJ, Rosner B, Willett WC, Speizer FE (1990). Risk factors for basal cell carcinoma in a prospective cohort of women. Ann Epidemiol.

[3] Hunter DJ, Maggwa BN, Mati JK, Tukei PM, Mbugua S (1994). Sexual behavior, sexually transmitted diseases, male circumcision and risk of HIV infection among women in Nairobi, Kenya. AIDS..

[4] Kapiga SH, Shao JF, Lwihula GK, Hunter DJ (1994). Risk factors for HIV infection among women in Dar-es-Salaam, Tanzania. J Acquir Immune Defic Syndr.

[5] Hunter DJ, Spiegelman D, Adami HO, Beeson L, van den Brandt PA, Folsom AR (1996). Cohort studies of fat intake and the risk of breast cancer—a pooled analysis. N Engl J Med.

[6] Smith-Warner SA, Spiegelman D, Yaun SS, van den Brandt PA, Folsom AR, Goldbohm RA (1998). Alcohol and breast cancer in women: a pooled analysis of cohort studies. JAMA.

[7] World Cancer Research Fund and American Institute for Cancer Research. Diet, Nutrition, Physical Activity and Cancer: A Global Perspective. Continuous Update Project Expert Report 2018. http://dietandcancerreport.org. Accessed 11 Mar 2019

[8] Wolff MS, Toniolo PG, Lee EW, Rivera M, Dubin N (1993). Blood levels of organochlorine residues and risk of breast cancer. J Natl Cancer Inst.

[9] Hunter DJ, Hankinson SE, Laden F, Colditz GA, Manson JE, Willett WC (1997). Plasma organochlorine levels and the risk of breast cancer. N Engl J Med.

[10] Laden F, Collman G, Iwamoto K, Alberg AJ, Berkowitz GS, Freudenheim JL (2001). 1,1-Dichloro-2,2-bis(p-chlorophenyl)ethylene and polychlorinated biphenyls and breast cancer: combined analysis of five U.S. studies. J Natl Cancer Inst.

[11] Chen J, Giovannucci E, Kelsey K, Rimm EB, Stampfer MJ, Colditz GA (1996). A methylenetetrahydrofolate reductase polymorphism and the risk of colorectal cancer. Cancer Res..

[12] Han J, Kraft P, Colditz GA, Wong J, Hunter DJ (2006). Melanocortin 1 receptor variants and skin cancer risk. Int J Cancer.

[13] Memisoglu A, Hu FB, Hankinson SE, Liu S, Meigs JB, Altshuler DM (2003). Prospective study of the association between the proline to alanine codon 12 polymorphism in the PPARgamma gene and type 2 diabetes. Diabetes Care.

[14] Easton DF, Pooley KA, Dunning AM, Pharoah PD, Thompson D, Ballinger DG (2007). Genome-wide association study identifies novel breast cancer susceptibility loci. Nature.

[15] Hunter DJ, Kraft P, Jacobs KB, Cox DG, Yeager M, Hankinson SE (2007). A genome-wide association study identifies alleles in FGFR2 associated with risk of sporadic postmenopausal breast cancer. Nat Genet.

[16] Wacholder S, Hartge P, Prentice R, Garcia-Closas M, Feigelson HS, Diver WR (2010). Performance of common genetic variants in breast-cancer risk models. N Engl J Med.

[17] Mavaddat N, Pharoah PD, Michailidou K, Tyrer J, Brook MN, Bolla MK (2015). Prediction of breast cancer risk based on profiling with common genetic variants. J Natl Cancer Inst.

[18] Campa D, Kaaks R, Le Marchand L, Haiman CA, Travis RC, Berg CD (2011). Interactions between genetic variants and breast cancer risk factors in the breast and prostate cancer cohort consortium. J Natl Cancer Inst.

